# The Rational Use of Disinfectants

**Published:** 1907-01-05

**Authors:** Thomas Divine

**Affiliations:** Fellow Roy. Inst. Pub. Health


### THE RATIONAL USE OF DISINFECTANTS.

By THOMAS DIVINE, M.D., C.M.(Glasg.), D.P.H. (Camb. and Lond.), F.C.S., Fellow Roy. Inst. Pub. Health.

Medical science in various ways attempts to combat our unseen foes, the ubiquitous bacilli; but whatever means of prophylaxis against disease be adopted, the need for efficient chemical disinfection will always exist. Chemical disinfection, therefore, must always take a high place among those measures to be employed against infection, both by the individual and by the community—by the citizen and by the health authorities. It is unfortunate that the conceptions of what constitutes infection and disinfection are still very chaotic. This state of affairs is due to the survival of antiquated and discredited notions, which imply that where the nose is not offended no danger exists. Modern bacteriological research has finally disposed of the fancy that infection is of the nature of a miasma, and has taught us to look upon it as due to a living entity—a micro-organism. The scientific conception of disinfection implies that the organism must be killed outright, and any agent which cannot thus kill the organism is no true disinfectant.

Heat, under certain definite conditions, can be depended upon as an efficient practical disinfectant. Whenever possible, infected clothing, fabrics, and such articles as will not spoil in water, should be disinfected either by prolonged boiling in water (preferably with some germicide of known efficiency), or by exposure in a steam disinfector under suitable physical conditions, and for a reasonable period of time. The modern tendency is towards the installation of apparatus giving saturated steam under pressure, as in the well-known Equifex. This is undoubtedly a move in the right direction, and a good disinfector of this type, even if the initial cost is high, is a necessity, and in the end an economy, in the equipment of a sanitary authority. As opposed to this, it may be safely affirmed that no kind of aerial disinfection can be absolutely depended upon. Koch, as long ago as 1881, showed the utter futility of pretending to disinfect with sulphur dioxide, either in the dry or moist condition, and the use of this agent has long been discarded on the Continent. More recently some authorities have pinned their faith to disinfection by formaline gas; but this, too, is untrustworthy against all but the least resistant organisms. Preparations of many chemical substances, such as permanganate of potassium, eucalyptus, thymol, boric acid, chloride of lime, and a host of proprietary articles, are erroneously believed by the public to be disinfectants. Some of them have the power of removing foul odours, or even in some measure of inhibiting bacterial multiplication, but these qualifications in no sense entitle them to be called disinfectants. They are at best nothing more than deodorants or antiseptics, and consequently their employment as disinfectants means a waste of money, and, what is even more to be regretted, tends to create a false sense of security.

In practice it is found that disinfection, apart from heat, is best accomplished by chemical agents of known efficiency and in solutions or suspensions of definite strength. In such a method contact is rapid, intimate, and certain, and the process tends to prevent dust rising to contaminate the surrounding atmosphere. But if disinfection by any chemical solution is to be a fact and not a farce, regard must be had to the special object in view. The power of each agent is not constant for different organisms; nor even for the same organism under different conditions.

The urgent need, therefore, of some means of "standardising" those chemical agents which are most frequently employed for the purpose of disinfection, is manifest, in the interest both of the public health and of municipal economy. By means of the Rideal-Walker method of standardising disinfectants we can now with certainty separate the efficient from the untrustworthy, the real from the spurious; and can award a figure of merit to each disinfectant in terms of a standard such as absolute phenol. Let us take, for example, two such well-known and largely used disinfectants as perchloride of mercury and the most active commercial carbolic acid, when tested against that common organism the typhoid bacillus. Regarding carbolic acid as the "standard," we find that the germicidal action of perchloride of mercury against the typhoid bacillus (the conditions in each case being the same), is 20 times as great as carbolic. In other words, the carbolic acid co-efficient of perchloride of mercury is 20. The cost of disinfecting by carbolic acid on the ordinary commercial price is 1s. per gallon, but the equivalent of this disinfecting power, as obtained by perchloride of mercury, would, on the same basis, be 1s. 3d. It will therefore be seen that this matter of efficiency and economy is one of no small importance. The need of some guarantee of the germicidal power of disinfectants will be apparent; and our contention is that it is desirable that the Legislature should call for a guarantee on all preparations which are sold to the public as disinfectants.

There are but few disinfecting substances on the market to which any guarantee in definite and scientific terms is attached. Such a guarantee attaches to the disinfectants prepared by Jeyes' Company, and more recently to the Izal of Newton, Chambers and Co., and to various preparations (Bactox, Sanitas, etc.) of the "Sanitas" Company. This is as it should be. For example, the well-known compound prepared by Jeyes' Company from certain members of a new series of oxidised hydrocarbons and sold under the name of Cyllin is guaranteed to be at least ten times less toxic than carbolic acid, and to have co-efficients, in terms of carbolic acid (absolute phenol), ranging from 10 to 30 for different micro-organisms. Thus against the common staphylococcus pyogenes aureus, Klein found the co-efficient of Cyllin to be 9.3; Firth and Rideal, and also Dunbar and Kister, found the co-efficient against the typhoid bacillus to be 11 (and since their work on this organism Messrs. Jeyes have succeeded in raising the co-efficient to 14); whilst Simpson and Hewlett give Cyllin the remarkable co-efficient of 35 against the bacillus pestis. It is not our present purpose to single out any particular germicide, but the scientific work performed with Cyllin alone illustrates the position we wish to take up—namely, that in the interest of public health, of efficiency, and of municipal economy, it is essential that regard must be had to the true germicidal power of any disinfectant employed. The Rideal-Walker method of standardising disinfectants can be applied to any disinfectant, and the appropriate figure of merit applied. When this is done one can by simple calculation rate the monetary cost of any germicide, and so have cost and efficiency placed side by side. In the case of Cyllin, for example, 3d. will purchase the germicidal power of 1s. worth of carbolic acid or 3s. 2d. worth of Lysol.

It is satisfactory to be able to record the fact that a score at least of the sanitary authorities in England and Wales are alive to the importance of having a definite guarantee as to the actual germicidal strength of the disinfectants they purchase with public moneys. We believe that the first public body in this country to insist on such a guarantee in connection with disinfectants was the Chelsea Borough Council.

We should like to see the co-efficient clause of the Metropolitan Borough of Islington (taken from the tender form, 1906) adopted by every municipality in this country: "Any disinfectant fluid may be tendered for provided that its bacteriological efficiency is expressed in terms of absolute phenol (100 per cent.) as determined by the Rideal-Walker method, when working with vigorous cultures of B. typhosus, and that it is miscible with water. The co-efficient must be given in the blank space left for the purpose."

There are many proprietary disinfectant substances capable, no doubt, of doing real service in disinfection when employed in suitable strength, but there are others which are quite useless; and the application of the Rideal-Walker figure of merit will place them at once on their proper plane of efficiency. But, until this is done, or until the Legislature steps in and demands a definite specification, neither private buyers nor public authorities have any effective control over the disinfectants they employ, and for all practical purposes they may be, and often are, buying and using disinfectants that will not disinfect.

In no other way can efficiency and substantial economy in our municipal expenditure on disinfectants be effected and a really rational system of chemical disinfection become established. We look to our health authorities all over the country to set their houses in order in this respect. They have officers who, by reason of their scientific training and experience, are capable of appreciating at their true worth the claims advanced for any disinfectant. It is by their medical officers of health, that local authorities should, in our opinion, be guided in the selection of disinfectants to be used on behalf of the community and of the public health.

